# Long-term outcomes of corneal intrastromal lenticule transplantation for necrotic scleral melting and glaucoma: A case report

**DOI:** 10.1097/MD.0000000000043490

**Published:** 2025-08-08

**Authors:** Jingyuan Yang, Bing Li, Gangwei Cheng

**Affiliations:** a Department of Ophthalmology, Peking Union Medical College Hospital, Chinese Academy of Medical Sciences, Beijing, China; b Key Laboratory of Ocular Fundus Diseases, Chinese Academy of Medical Sciences, Beijing, China.

**Keywords:** case report, corneal intrastromal lenticule, glaucoma, scleral melting, surgery

## Abstract

**Rationale:**

Necrotic scleral melting presents a significant therapeutic challenge, especially when complicated by concurrent conditions like refractory glaucoma. Conventional repair materials often have limitations. This case report aimed to evaluate the long-term efficacy and safety of a novel approach: utilizing an allogeneic corneal intrastromal lenticule (typically discarded after small incision refractive lenticule extraction surgery) combined with an autologous conjunctival graft for repairing necrotic scleral melting in a complex patient with glaucoma. We specifically investigated the lenticule’s potential for structural integration and its surprising role in aqueous humor drainage.

**Patient concerns:**

A 29-year-old male with a history of congenital cataract surgery and multiple surgeries for retinal detachment and secondary glaucoma presented with necrotic scleral melting at the filtration site of a previous glaucoma surgery. The intraocular pressure was low due to the damage to the integrity of the eyeball.

**Diagnosis and intervention:**

Necrotic scleral melting is diagnosed. Surgical repair was performed using an allogeneic corneal intrastromal lenticule, extracted during small incision refractive lenticule extraction surgery, combined with an autologous conjunctival graft.

**Outcomes:**

At the 36-month follow-up, the globe remained structurally intact. Intraocular pressure stabilized within the normal range (9–14 mm Hg) without any hypotensive medication, significantly improved from the preoperative hypotony (5 mm Hg). Visual acuity improved from light perception to counting fingers. Imaging (optical coherence tomography, ultrasound biomicroscopy) demonstrated successful fusion of the lenticular graft with the surrounding scleral tissue and revealed the formation of a functional aqueous drainage channel within the supraciliary space. No rejection, infection, or graft melting occurred during the entire follow-up period.

**Lessons:**

Corneal intrastromal lenticules may represent a novel and accessible solution for the treatment of scleral melting, offering not only effective structural repair but also functioning as a filtration pathway. This material demonstrated good long-term safety and efficacy, making it a promising option for managing complex scleral conditions.

## 1. Introduction

Necrotic scleral melting is a severe open globe injury due to various causes. The main therapeutic principle is to recover the closure as soon as possible. Various materials, such as amniotic membranes and scleral grafts, have been tried on this issue. Treatment of scleral melting will be more difficult when combined with other diseases, such as refractory glaucoma.^[1]^ Optimized materials and surgical methods have been investigated due to unsatisfactory success rates.

Allogeneic cornea lenticules collected from routine small incision refractive lenticule extraction (SMILE) surgeries have been used to treat corneal ulcers and perforations. However, it remains unknown whether the lenticules could be utilized for scleral perforations and other complex situations.

Here, we report the 3-year long-term outcomes of a case of necrotic scleral melting and refractory glaucoma treated with intrastromal corneal lenticule and conjunctival autograft transplantation and found that the lenticule could fuse to adjacent scleral tissue and allow humor to flow through. The case report was approved by the Institutional review board of Peking Union Medical College Hospital (S-K631) and written informed consent was obtained from the patient.

## 2. Case presentation

A 29-year-old male presented with decreased vision and a sense of streaming in the right eye. The visual acuity was only light perception. In the past 3 years, he was diagnosed with glaucoma, secondary to recent scleral buckling and vitrectomy for retinal detachment. Filtration surgery, drainage device implantation, ultrasound cycloplasty, and cyclophotocoagulation were performed sequentially for glaucoma. He was also performed phacoemulsification and intraocular lens implantation in bilateral eyes due to congenital cataract at 3. The intraocular lens in the right eye was extracted at the last vitrectomy in 2019 for the treatment of anterior proliferative vitreoretinopathy, and the retina was reattached completely.

**Figure 1.**
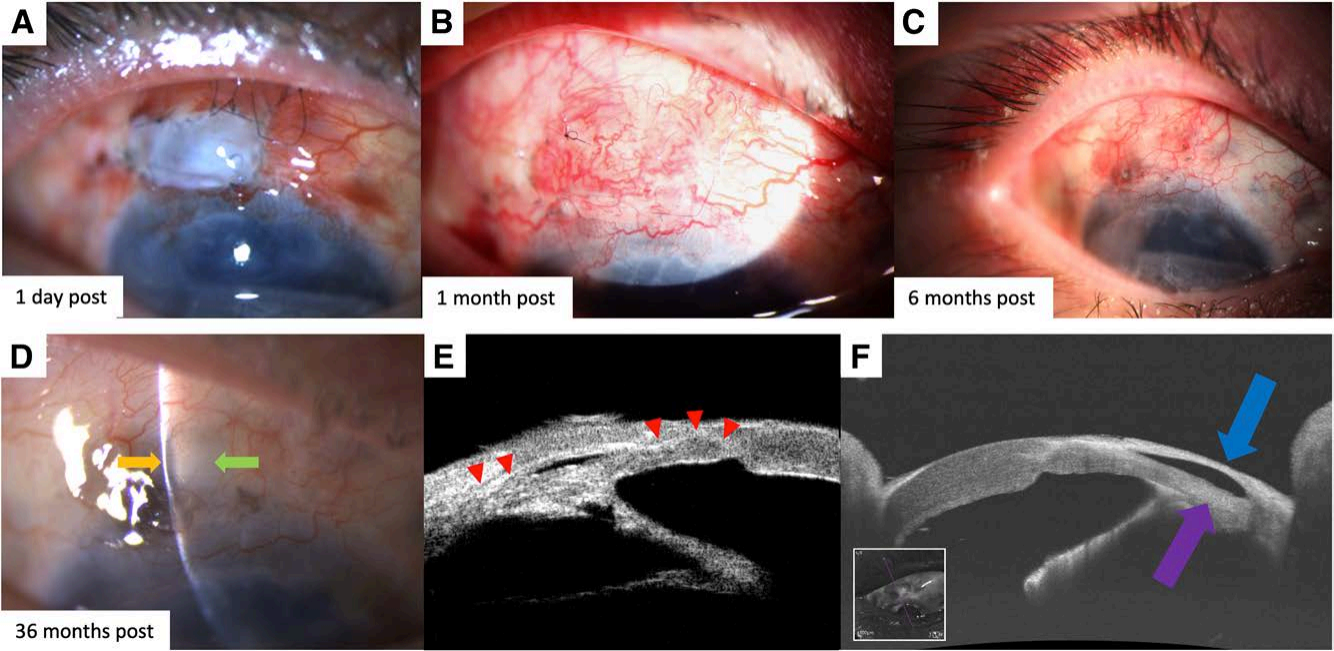
The images of the 36-month follow-up. (A–D) One day postoperatively, the corneal lenticule graft and the conjunctival graft were well located, and the eyeball shape was maintained. 36 months after the surgery, the superior eyeball was integrity, and the conjunctiva was smooth and flattened. The translucent gray-white graft of intrastromal corneal lenticule under the filtration bleb can be noticed (green arrow), with no neovascularization. (E) The edge of the lenticular graft was blurring and fused with surrounding tissue (red arrowhead) in 6 months. (F) OCT image of the lesion area 3 years after the surgery shows that the corneal intrastromal lenticule, which is slightly hyperreflective, has fused with the surrounding tissue (purple arrow). The subconjunctival filtering bleb is maintained well (blue arrow). OCT = optical coherence tomography.

The eyeball was deformed due to extremely hypotony, and we performed emergency surgery. A sclera fistula of approximately 2 × 1mm was found at the superior limbus beneath the contracted conjunctival cicatrization, which resulted from the melting of the anterior portion of the sclera lamellar bed created at the prior filtration surgery. Necrotic scleral melting was confirmed during the surgery. After careful dissection of the scarring conjunctiva and removal of the exposed intraocular part of Ahmed glaucoma valve, an allogeneic cornea lenticule collected from a routine SMILE surgery was pruned from a 6 mm circle to a 4 × 3mm rectangle and stitched interruptedly with 1 mm interval using 10-0 nylon sutures to the adjacent healthy sclera to cover the fistula. An autograft of conjunctiva from the inferotemporal quadrant was transplanted to repair the superficial conjunctiva (Fig. 1A).

The eyeball regained its integrity (Fig. 1A–D). Intraocular pressure (IOP) rose from 5 mm Hg one day postoperatively to 14 mm Hg 6 months after without hypotensive agents and gradually lowered to 9 mm Hg at an 36-month visit. Visual acuity improved from light perception to counting finger in 6 months, comparable to pre-op level. Optical coherence tomography (OCT) and ultrasound biomicroscopy (UBM) showed the lenticular graft located well and fused to surrounding tissue (Fig. 1E, F). A drainage channel in the supraciliary space has formed 6 months postoperatively (Fig. 1E). No rejection reaction or other complications were observed during the 36-month follow-up (Fig. 1D).

## 3. Discussion

In this case, we reported satisfied 36-month long-term outcomes of using a corneal intrastromal lenticule graft for necrotic scleral melting and glaucoma, and the graft fused with adjacent tissue and worked as an unexpected drainage channel in the supraciliary space. Although we performed conventional surgery procedures, we used novel transplantation materials of intrastromal corneal lenticule and conjunctival autograft.

Our primary finding is that integration of the grafted piece with surrounding tissue and control of related diseases are important success factors when treating similarly complex conditions, which has been proved by examinations (e.g. UBM and IOP measurement) in the long term. Expected outcomes of the surgery include not only closure of the opening but also long-term control of IOP. For the patient, no ocular agent was needed to sustain IOP postoperatively. The fusion of lenticular graft with surrounding sclera on UBM and OCT images as a good prognostic factor might benefit from limbus blood supply and good histocompatibility. We speculated that the water permeability of corneal lenticule and fibrillation between the graft and sclera tissue preserved the filtration and outflow of aqueous humor. Recover of limbus and conjunctiva might also facilitate the humor outflow.^[2]^ This hypothesis provides a potential novel solution to refractory glaucoma, which still needs further observation and investigation. Moreover, an unexpected drainage channel detected using OCT and UBM also facilitated the humor outflow.

The materials of allogeneic intrastromal corneal lenticule and conjunctival autograft are easy to obtain for scleral melting nowadays. Longitudinal visits revealed a good tolerance to lenticular allograft and long-term safety. The material of intrastromal corneal lenticule from SMILE is available in most ophthalmic centers. The reusage of intrastromal corneal lenticule has been reported to treat cornea perforation due to its immune privilege, good histocompatibility, and tolerance to strength and pressure.^[3]^ However, its long-term effectiveness and safety for scleral melting have not been reported previously. No obvious inflammation or reaction of rejection was noticed during the 36-month follow-up. As the urgent requirement of repairment, intrastromal corneal lenticule, as materials much easier to acquire than other materials (e.g., pericardium patch graft), could be investigated and used in more similar cases.^[4]^

This study reports the outcomes of a single case. While the 3-year follow-up provides valuable long-term data, the findings require validation in larger cohorts with diverse presentations of scleral melting. The unique success observed here might be influenced by factors specific to this patient, such as the location of the melting at the limbus and the prior surgical history. The mechanism by which the lenticule facilitated aqueous outflow warrants further histological and physiological investigation. Despite these limitations, the promising long-term results suggest corneal lenticules warrant consideration as a viable option for complex scleral repair.

## 4. Conclusion

Our present therapy highlights the method’s long-term outcomes and related success factors. We recommend transplantation of corneal lenticule grafts with conjunctival autografts for urgent eyeball rupture as a novel and safe solution. The corneal intrastromal lenticules could serve as a repair material and a filtering channel.

## Acknowledgments

The authors thank the patient and all the clinical staff who participated in the treatment of the patient.

## Author contributions

**Conceptualization:** Jingyuan Yang, Gangwei Cheng.

**Methodology:** Jingyuan Yang, Bing Li, Gangwei Cheng.

**Writing – original draft:** Jingyuan Yang.

**Writing – review & editing:** Jingyuan Yang, Bing Li, Gangwei Cheng.
